# Unraveling quantitative trait loci (QTL) overlapping epigenomic regulatory regions associated with feeding behavior in pigs

**DOI:** 10.3389/fgene.2026.1779847

**Published:** 2026-04-10

**Authors:** Izally Carvalho Gervásio, Luiz F. Brito, Andre C. Araujo, Simara Larissa Fanalli, Bárbara Silva-Vignato, Artur O. Rocha, Lorena Ferreira Benfica, Leticia Fernanda de Oliveira, Andrezza Maria Felício Ament, Gabriel Costa Monteiro Moreira, Cristina Tschorny Moncau-Gadbem, Aline Silva Mello Cesar

**Affiliations:** 1 Department of Food Science and Technology, Luiz de Queiroz College of Agriculture (ESALQ), University of São Paulo, São Paulo, Brazil; 2 Department of Animal Sciences, Purdue University, West Lafayette, IN, United States; 3 AcuFast Swine, Saskatoon, SK, Canada; 4 College of Animal Science and Food Engineering (FZEA), University of São Paulo, São Paulo, Brazil; 5 Institut National de Recherche pour l’Agriculture, l’Alimentation et l’Environnement (INRAE), Igny, Île-de-France, France; 6 Faculty of Veterinary Medicine and Animal Science (FMVZ), University of São Paulo, São Paulo, Brazil

**Keywords:** functional genomics, genetic markers, integrative genomics viewer genome browser, pig breeding, SNP mapping

## Abstract

**Introduction:**

Feed behavior traits are directly linked to the sustainability of pig production. This study aimed to investigate the genetic basis of feeding behavior traits in Landrace and Yorkshire pigs, focusing on genomic regions, quantitative trait loci (QTL), candidate genes, and metabolic pathways associated with these traits.

**Methods:**

Genome-wide association studies (GWAS) were performed, followed by functional enrichment analyses and integrative bioinformatics approaches to identify biologically relevant genomic regions, candidate genes, and pathways associated with feeding behavior traits.

**Results:**

Several candidate genes and transcription factors were identified, including STAT3, STAT5A, STAT5B, RARA, SMAD4, MYC, FOXP3, SP1, NOTCH1, and RXRA. In addition, strong candidate genes such as SLC22A2, CPAMD8, and NKX2-6 were highlighted as potentially influencing feeding behavior traits.

**Discussion:**

These findings improve the understanding of the genomic architecture underlying feeding behavior traits and provide valuable insights for the development of more efficient breeding programs, contributing to improved animal production efficiency and welfare.

## 1 Introduction

A comprehensive understanding of the genetic factors influencing feeding behavior and feed efficiency traits is essential for developing and refining genetic and genomic selection strategies to enhance the sustainability of animal production systems (Do et al., 2013). Improving feed efficiency is crucial for reducing production costs and the environmental impact of pig farming, as it directly reflects nutrient allocation for growth and maintenance (Patience et al., 2015). Feeding behavior traits, including the frequency and duration of feeder visits, directly correlate with an animal’s ability to consume and digest nutrients efficiently (Patience et al., 2015). These traits significantly impact economic metrics in pig production, such as weight gain and feed conversion efficiency (Canario et al., 2020).

Transcription factors are key regulators of gene expression and play essential roles in controlling biological processes, including the modulation of feeding behavior traits in pigs (Zhang et al., 2025). By binding to specific DNA sequences, they activate or repress the transcription of genes essential for metabolism and energy homeostasis (Piórkowska et al., 2019). Understanding the role of these factors in gene promoters and enhancers can provide insights into the molecular mechanisms underlying economically relevant phenotypes, such as feed intake and feed efficiency (Li et al., 2025).

The identification of quantitative trait loci (QTL) has been essential in clarifying the genetic basis of complex traits related to feeding behavior. QTL corresponds to genomic regions that contain variants associated with phenotypic variation of a complex trait and may encompass regulatory genes and epigenetic elements (Ibragimov et al., 2022). The integration of QTL with epigenetic information can enable the identification of candidate genes and biological pathways involved in regulating feed intake and efficiency (Shi et al., 2025). Investigating QTL offers valuable insights into the genetic mechanisms that influence these traits, potentially leading to more effective genetic selection and animal breeding strategies (Gonçalves da Silva et al., 2025).

Chromatin states represent distinct functional configurations of the genome, defined by combinatorial patterns of epigenetic marks. These epigenetic modifications, such as DNA methylation and histone alterations, regulate chromatin architecture and DNA accessibility to transcription factors. Consequently, the resulting chromatin state determines whether a genomic region is transcriptionally active, poised, or repressed across different tissues and physiological conditions (van der Velde et al., 2021). Recent studies suggest that SNPs linked to feeding behavior are often found in areas with active chromatin states, highlighting the need to integrate genomic and epigenomic data to better understand the underlying regulatory mechanisms (Li et al., 2021). Therefore, analyzing chromatin states can contribute to the establishment of a strong foundation for future research on tissue-specific gene regulation and the development of more precise genomic selection strategies (Grubert et al., 2015). Therefore, the main objectives of this study were to identify QTL associated with pig-feeding behavior and to characterize epigenomic regulatory regions, candidate genes, pathways, and transcription factors within or near these QTL.

To achieve our goals, the present study utilized a multifaceted and integrative approach to investigate the genetic and regulatory landscape underlying feeding behavior traits in pigs, specifically focusing on AFIV and AOTD. First, GWAS was used to identify associated QTL. Then, we characterize the epigenomic regulatory regions underlying these traits and identify candidate genes, metabolic pathways, and transcription factors within or near the associated QTL. SNP annotation; assessment of overlap with known QTL; functional enrichment analysis of relevant genes; transcription factor association analysis; and integration of chromatin state data using IGV for visualization were the methods utilized. This multifaceted approach, incorporating chromatin state data and allowing for the visualization of SNPs within specific chromatin environments, provided a comprehensive view of the regulatory landscape influencing feeding behavior. Moreover, this integrated analysis of GWAS, epigenomic data, and functional annotations facilitated the identification of key molecular mechanisms and candidate genes, ultimately aiming to contribute to improved genetic selection strategies in pig production by furthering our understanding of the influence of these QTL on AFIV and AOTD in pigs. Feeding behavior is a complex trait influenced not only by genetic variation but also by epigenomic mechanisms and environmental stimuli. Epigenetic modifications, such as DNA methylation and histone acetylation, can regulate the expression of genes involved in appetite control, reward pathways, and energy homeostasis, without altering the DNA sequence itself. These modifications are often sensitive to external factors, including diet composition, feeding routines, social hierarchy, and environmental stressors. Thus, environmental inputs may lead to long-lasting changes in gene expression through epigenomic reprogramming, particularly during early developmental stages or periods of nutritional transition. Recognizing the interplay between genetic architecture, epigenomic regulation, and environmental influences is essential to better understand variability in feeding behavior and to improve strategies for genetic selection and animal management.

## 2 Materials and methods

### 2.1 Data

Approval from the Animal Care Ethics Committee was not required for the present study, as all records were obtained from data routinely collected under commercial production conditions. This study was based on a retrospective observational dataset derived from nucleus herds of a North American pig breeding company. Phenotypic, genotypic, and pedigree information were available for 9,023 male Landrace and 12,166 male Yorkshire pigs evaluated between January 2016 and December 2022. All analyses were performed separately for each breed. Each animal was evaluated during a 80 days (± 2 weeks) feed intake test conducted on a nucleus farm, entering the trial at approximately 30 kg body weight and remaining under evaluation until reaching approximately 120 kg. This period corresponds to the growing–finishing production phase, during which feeding behavior traits are considered biologically stable and relevant for feed efficiency evaluation.

In the test, pigs were allocated to 56 pens containing 12 animals each, with pens composed of animals from the same breed. Feed intake and feeding behavior were individually recorded using Feed Intake Recording Equipment (FIRE; Osborne Industries Inc., Osborne, KS, United States), which allows continuous 24-h access to feed while permitting only one animal at a time to access the feeder, enabling individual intake measurements under group housing conditions. Each pig was equipped with an individual electronic identification transponder (RFID tag), which allowed the FIRE system to automatically recognize the animal at each feeder visit. This system records individual feeding events, including visit time, duration, and feed intake, ensuring precise allocation of measurements to each animal.

Feed intake data were collected under two distinct recording schemes: continuous recording summarized weekly and intermittent recording summarized bi-weekly. Each recording scheme involved different groups of animals evaluated during the experimental period. In the weekly scheme, feeding behavior and feed intake were continuously recorded from the first to the last day of the test period. In the bi-weekly scheme, data were collected intermittently, following alternating periods of 7 days of recording and 7 days without recording, resulting in fewer observations per animal compared to the continuous regime. To increase sample size and statistical power for genome-wide association analyses, the weekly and bi-weekly datasets were merged into a single combined dataset (ALL dataset). Thus, the ALL dataset represents independent animals evaluated under comparable management conditions but different recording schedules. In all analyses, the animal was considered the experimental unit, with repeated observations collected throughout the approximately 80-day feed efficiency test period.

### 2.2 Feeding behavior traits

Five feeding behavior traits were analyzed in this study: 1) average daily occupancy time (AOTD), which represents the average amount of time (in seconds) that an animal spent at the feeder per day; 2) average number of daily visits to the feeder, i.e., the average number of visits an animal made to the feeder units (ANVD, in counts); 3) average feed intake per visit to the feeder during the test period (AFIV, in grams); 4) average occupancy time (in seconds) per visit to the feeder during the test period (AOTV); and, 5) average feed rate per visit during the test period (AFRV, in grams/minutes), which represents the average amount of feed an animal consumed per minute of each visit to the feeder units (Lu et al., 2017).

### 2.3 Pedigree data

The pedigree datasets provided detailed information on the genetic structure of the studied populations. The Yorkshire pedigree database contains 477,359 animals, including 3,747 sires and 40,125 dams. The Landrace pedigree contained 304,166 individuals, including 2,497 sires and 19,517 dams.

### 2.4 DNA extraction/genotyping

Tissue samples used for genotyping consisted of ear tissue collected at birth during routine litter processing using a standard Tissue Sampling Unit (TSU). The sampling procedure followed standard commercial pig production practices, in which ear tissue is collected simultaneously with animal identification, minimizing animal handling and stress. Genomic DNA was extracted from these samples and subsequently used for SNP genotyping.

### 2.5 Genomic data

A total of 50,000 animals (25,000 males per breed) were genotyped using the FastGen1 SNP panel (AcuFast, Saskatoon, Saskatchewan, Canada). The FastGen1 panel is a commercially available imputed SNP panel developed from customized 50K, 60K, and 70K SNP arrays, in which genotypes are pre-imputed by the company prior to data delivery. Specifically, a subset of animals (e.g., individuals born in earlier cohorts such as 2015) had their genotypes imputed by the company before inclusion in the database. No additional genotype imputation was performed in the present study. All marker positions were updated to the pig reference genome assembly Sscrofa11.1 (https://www.ncbi.nlm.nih.gov/datasets/genome/GCF_000003025.6/).

Before quality control (QC), the Landrace and Yorkshire genomic datasets contained 43,873 and 46,018 SNPs, respectively. Quality control was performed using PREGSF90 (Misztal et al., 2014). Markers with call rate <0.95, minor allele frequency (MAF) < 0.05, and deviations between observed and expected heterozygosity greater than 0.15 were removed. Individuals with genotyping call rate <0.95 were also excluded. After QC, 41,060 SNPs for 24,861 Landrace pigs and 44,906 SNPs for 24,947 Yorkshire pigs remained for further analyses.

To ensure computational feasibility while maintaining population representativeness, a subset of genotyped animals was selected for GWAS analyses. The sampling strategy included all animals with available phenotypic records, their parents with phenotypic information, and a random sample of the remaining genotyped individuals. The selection procedure considered a broader pool of genotyped animals from the commercial population (69,338 Landrace and 101,651 Yorkshire pigs born between 2016 and 2022) to preserve population structure across birth years and maintain pedigree connectivity. The final subset (∼25,000 animals) was therefore representative of the population while retaining genetic relationships among animals with known phenotypes.

### 2.6 Genome-wide association studies (GWAS)

We performed a single-step genome-wide association study (ssGWAS) using the BLUPF90+ family of programs (Misztal et al., 2022). First, we estimated variance components and calculated the genomic estimated breeding values (GEBVs) for the five feeding behavior traits. Subsequently, POSTGSF90 was used to back-solve GEBVs into SNP effects and calculate their approximate *p*-values. Phenotypes were not adjusted for body weight. The statistical model used to evaluate the traits was:

y=Xβ+Za+Wp+e

where y, β, a, p, and e are vectors of phenotypic observations, fixed effects (the contemporary group (CG) was defined by combining the year of the test, the test week, and the feeder used, ensuring that animals compared within a group experienced similar environmental and management conditions). Additionally, age at entry to the test was fitted as a linear covariate to account for differences in developmental stage among animals, random additive genetic effects, permanent environmental effects, and residual effects, respectively. X, Z, and W are the incidence matrices for fixed, animal additive genetic, and permanent environmental effects, respectively. The animal, permanent environmental, and residual effects were considered random, and there were repeated records for all five traits. In the univariate model (single traits analyses), we assumed a∼N(0, Hσ2a), *a∼N0, Hσa2,* p ∼ N(0, Iσ2p)*p ∼ N(0, Iσp2)* and e ∼ N(0, Iσ2e)*e ∼ N(0, Iσe2)*, where σ2a, σ2p, and σ2e are the additive genetic, permanent environment, and residual variances, respectively. H is a hybrid genomic relationship matrix (Misztal et al., 2022), and I is an identity matrix.

Body weight was not included as an additional covariate in the GWAS model because phenotypic records had been previously corrected using the approach proposed by Casey et al. (2003), Casey et al. (2005), in which body weight was fitted as a linear covariate during phenotypic data editing and correction. Therefore, the GWAS analyses were performed using already adjusted phenotypes, preventing double adjustment effects.

**FIGURE 1:**
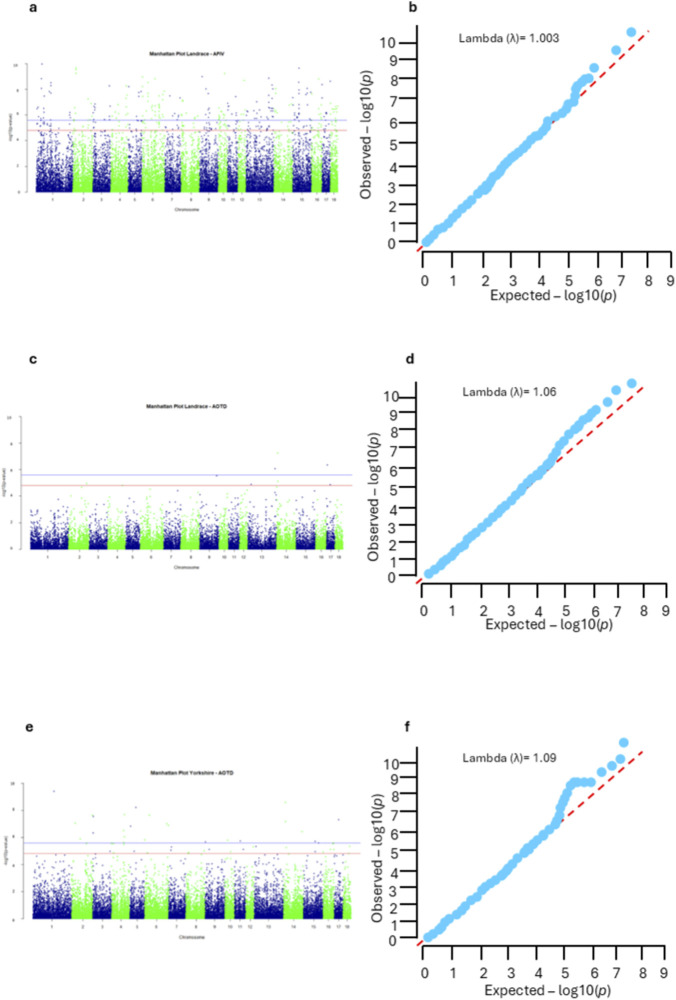
Manhattan and quantile-quantile (QQ) plots of genome-wide association study results for feeding behavior traits in pigs. **(a)** Manhattan plot and **(b)** QQ plot for average feed intake per visit (AFIV) in Landrace pigs. **(c)** Manhattan plot and **(d)** QQ plot for average daily occupancy time (AOTD) in Landrace pigs. **(e)** Manhattan plot and **(f)** QQ plot for AOTD in Yorkshire pigs. In Manhattan plots **(a)**, **(c)**, and **(e)** the solid blue lines indicate the Bonferroni-corrected significance thresholds: log_10_(p) = 5.91 for Landrace and–log_10_(p) = 5.95 for Yorkshire pigs. The red dashed lines represent suggestive significant thresholds.

A quantile–quantile (Q–Q) plot was used to compare the distribution of observed p-values with the expected uniform distribution under the null hypothesis of no association. Additionally, the genomic inflation factor (λ) was calculated based on the median of the observed statistical test to evaluate potential deviations from the expected null distribution, which may arise from confounding factors such as population structure or relatedness. The λ values ranged from 1.003 to 1.09 (Figure 1).

Genome-wide significance thresholds were established using the standard Bonferroni correction to control the family-wise error rate (FWER), adjusting the significance level according to the number of SNPs tested in each breed. For the Landrace population (with 41,060 SNPs), the genome-wide significance threshold was set at −log10(0.05/N) = 5.91, and the suggestive threshold at −log10(1/N) = 4.61, where N = 41,060 SNPs, respectively. For the Yorkshire dataset with 44,906 SNPs, the significance threshold was −log10(0.05/N) = 5.95, and the suggestive threshold was set to −log10(1/N) = 4.65, where N = 44,906 SNPs, respectively. For downstream analyses, only SNPs exceeding the genome-wide significance threshold (Bonferroni-corrected, 0.05/N) were considered, ensuring that subsequent analyses were based on statistically robust associations.

### 2.7 Functional annotation and data integration

A comprehensive analysis and interpretation of the GWAS results was conducted using a range of bioinformatics tools. The Ensembl BioMart (Ensembl Variation v113) was employed to retrieve rs IDs (Reference SNP cluster IDs–Sherry et al., 2001) corresponding to the significant SNPs identified. Additionally, functional annotation of these variants was carried out using the Ensembl Variant Effect Predictor (VEP) to characterize their potential biological consequences. Despite the original moderate SNP density, our imputed dataset, combined with ±500 kb windows, ensures reliable functional annotation of potentially relevant variants. Genes overlapping relevant SNP windows were identified using the Ensembl Genes database v113 and the *Sus scrofa* genome v11.1 (Warr et al., 2020) as reference. The DAVID v2024q2 database (Dennis et al., 2003; Huang et al., 2009) was used for functional enrichment analyses of those genes. Only Gene Ontology (GO) terms and Kyoto Encyclopedia of Genes and Genomes (KEGG) pathways with corrected P-values less than 0.05 (Benjamini–Hochberg correction) were considered significantly enriched. The target region refers to the genomic segment surrounding each significant SNP, which was considered for downstream functional annotation and candidate gene identification analyses. In our study, the target region was defined by extending 500 kb upstream and downstream of each significant SNP. This genomic window size was chosen based on the typical linkage disequilibrium (LD) decay in pigs (Ai et al., 2015; Grossi et al., 2017). By using this genomic window, we aimed to capture not only the significant SNP itself but also nearby regulatory elements and genes that could be influenced by or linked to the identified variant. This approach is particularly useful when working with medium-density SNP chips, such as the imputed FastGen1 array, where SNP spacing may not directly capture all functional sites.

Protein–protein interaction (PPI) enrichment analysis was performed using the Enrichr platform (Chen et al., 2013; Kuleshov et al., 2016) to evaluate whether genes located within GWAS-associated regions are functionally connected at the protein level. This analysis was used as a post-GWAS functional interpretation approach to identify potential biological networks and shared molecular pathways underlying feeding behavior traits, rather than to infer the physical location or functional effects of individual SNPs. Additionally, transcription factor enrichment analyses were conducted at the gene level with the purpose of elucidating gene expression regulation, rather than evaluating the direct disruption of transcription factor binding sites by individual SNPs. Subsequently, to investigate regulatory QTL, we performed QTL annotation using the GALLO v1.5 R package (Fonseca et al., 2020) to annotate genomic regions associated with feeding behavior traits. The analysis was based on QTL data retrieved from the Pig QTL database (Hu et al., 2022) to ensure comprehensive coverage of genomic regions associated with economically relevant traits in pigs. Finally, we integrated GWAS results with chromatin state predictions from Pan et al. (2021) using the Integrative Genomics Viewer (IGV) to identify regulatory regions influencing pig feeding behavior. Chromatin accessibility and histone modification profiles provided insights into the regulatory landscape of candidate regions. For this analysis, we specifically explored chromatin state data from muscle, adipose, cortex, and liver tissues, as these tissues play key roles in the biological regulation of feeding behavior and energy metabolism. The cortex and hypothalamic tissues were included due to its involvement in neural processes associated with appetite control, decision-making, and reward-related feeding responses (Malik et al., 2008). Liver and adipose tissues were selected because of their central roles in metabolic regulation, nutrient sensing, and energy homeostasis, which influence feeding motivation and efficiency (Rui, 2014; Ahima and Flier, 2000). Muscle tissue was considered given its relevance to energy utilization and metabolic demand, traits closely linked to feeding patterns and efficiency in pigs (Wolfe, 2006). The inclusion of these tissues allowed the functional interpretation of associated genomic regions within biologically relevant regulatory contexts. While hypothalamic tissue was included in our analyses, we did not observe statistically significant associations with feeding behavior traits in this tissue. The complete employed datasets are available for public access via the following links: https://pigbiobank.farmgtex.org/ (Zeng et al., 2024) and https://figshare.com/articles/dataset/6_type_of_regulator_hg19_zip/13480425 (Pan et al., 2021).

## 3 Results

### 3.1 Descriptive statistics of feeding behavior traits

Descriptive statistics for feeding behavior traits are presented for both breeds. In the Landrace population, the average feed intake per visit (AFIV) was 382.68 g (SD = 184.73), ranging from 120.00 to 900.00 g, based on 2,996,649 records collected from 9,023 animals. The average occupation time per day (AOTD) showed a mean value of 143.08 s (SD = 74.76), with values ranging from 54.00 to 599.65 s using the same number of records and animals. In the Yorkshire population, AOTD presented a mean of 130.43 s (SD = 77.35), ranging from 24.00 to 598.55 s, based on 3,837,583 records obtained from 12,166 animals. Overall, the observed variability in feeding behavior traits indicates substantial phenotypic variation within breeds, supporting their suitability for genome-wide association analyses.

### 3.2 Genome-wide association analyses

The GWAS results for feeding behavior traits revealed no significant SNPs for AFRV, ANVD, and AOTV traits in the Landrace breed after multiple testing corrections. Similarly, no significant SNPs were associated with AFIV, AFRV, ANVD, and AOTV traits in the Yorkshire population (Supplementary Figure S1).

In the present study, only AFIV and AOTD feeding behavior traits were found to be significantly associated with the tested genomic database. For the Landrace, 186 SNPs distributed across the genome (all autosome chromosomes) were found to be associated with AFIV. In comparison, variants associated with AOTD in Landrace were concentrated on chromosomes SSC13, SSC14, and SSC17 (three significant SNPs). For Yorkshire, 22 SNPs associated with AOTD were broadly distributed across chromosomes (SSC1, SSC2, SSC3, SSC4, SSC5, SSC6, SSC10, SSC11, SSC14, SSC15, and SSC17). Figure 1 presents the Manhattan and QQ plots showing these associations, and the complete list of significant SNPs can be found in Supplementary Table S1. The windows that explained more than 1% of the total additive genetic variance for each feeding behavior trait are summarized in the Supplementary Table S2. Estimation of genetic parameters was not the objective of the present study, which focused on identifying genomic regions associated with feeding behavior traits through GWAS.

### 3.3 SNP annotation

The GWAS-identified significant SNPs were subjected to VEP analysis to ascertain their potential functional and positional effects on the genome. The significant SNPs were in coding, regulatory, and intergenic regions, as can be seen in Supplementary Tables S3, S4. Beyond characterizing SNP consequences, this analysis also identified the genes in which these variants are located, pinpointing potential candidate genes that influence feeding behavior.

For Landrace pigs, the 186 significant SNPs associated with AFIV were harbored in 114 unique genes, of which 55 were related with multiple SNPs, indicating potential pleiotropic or recurrent regulatory effects. About the coding consequences, 3.0% were in coding regions, of which 0.8% were missense variants, while 2.2% were predicted as synonymous variants. The genomic regions identified included intronic regions (58.5%), intergenic regions (19.7%), downstream gene regions (5.1%), and regulatory regions (enhancers and open chromatin regions, 3.8%) (Supplementary Figure S2A). Candidate genes included myosin VB (*MYO5B*), collagen type XXVII alpha 1 chain (*COL27A1*), solute carrier family 22 member 2 (*SLC22A2*), cell adhesion molecule 2 (*CADM2*), U6 spliceosomal RNA, a small nucleolar RNA (snoRNA), and several long noncoding RNAs (lncRNA). Of the three SNPs associated with AOTD in Landrace, those located on SSC13 (rs345929299) and SSC17 (rs327605041) were predicted to be intergenic. Interestingly, the SNP on SSC14 (rs81305085) within the *NKX2-6* gene (NK2 homeobox 6) was a missense variant, although its SIFT (Sorting Intolerant from Tolerant) score was predicted as tolerated (SIFT score = 0.53), indicating that it not affect protein function (Sim et al., 2012; Ng and Henikoff, 2003) (Supplementary Figure S2B).

In Yorkshire pigs, the 22 SNPs associated with AOTD were harboring 15 unique genes, from those 11 genes were related with more than one SNP. About the variant consequences, 11.3% exhibited coding consequences, including 1.9% missense. The identified genomic locations included intronic regions (52.8%), intergenic regions (15.1%), 3′UTR regions (7.5%), and downstream gene regions (5.7%). Regulatory regions (enhancers) contained 9.4% of the variants, with an additional 3.8% located at splice sites (Supplementary Figure S2C). Candidate genes included calpain-2 (*CAPN2*), apoptosis-stimulating protein 2 of p53 (*TP53BP2*), acyl-CoA synthetase short-chain family member 1 (*ACSS1*), ankyrin repeat domain containing 11 (*ANKRD11*), and a novel lncRNA on SSC1. The unique missense variant (rs329447474) located on SSC2 within the *CPAMD8* gene (complement component 3- and pregnancy zone protein-like alpha-2-macroglobulin domain-containing protein 8), presents a SIFT score = 1, also indicating that it not affect protein function. To further support the biological relevance of prioritized loci identified during SNP annotation, genotype-phenotype comparisons were performed for representative variants.

### 3.4 Genotype-phenotype comparisons

**FIGURE 2:**
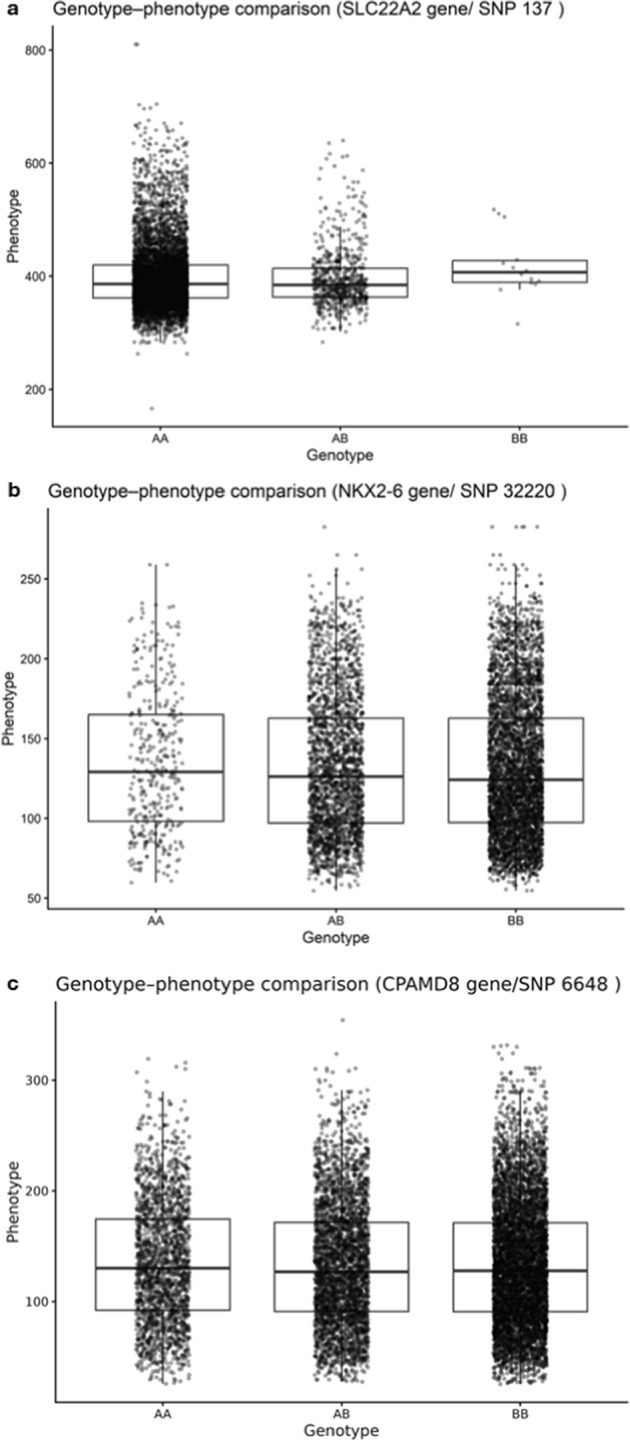
Genotype-phenotype comparisons for representative SNPs associated with feeding behavior traits. Boxplots represent phenotypic distributions across genotypic classes (AA, AB, BB) for **(a)** SNPs located near *SLC22A2* (AFIV (grams) in Landrace), **(b)** *NKX2-6* (AOTD (seconds) in Landrace), and **(c)** *CPAMD8* (AOTD (seconds) in Yorkshire). Points indicate individual observations, boxes represent interquartile ranges, and error bars denote standard error of the mean.

To facilitate biological interpretation of prioritized loci, genotype-phenotype comparisons were performed for representative SNPs located near candidate genes associated with feeding behavior traits (Figure 2). Differences among genotype classes reflect genotype-specific phenotypic means rather than overall descriptive averages, as values represent conditional means within each genotype group. For AFIV in Landrace pigs, the SNP located near *SLC22A2* gene (SNP position: 137) showed differences in average feed intake per visit among genotypic classes. Animals carrying the AA genotype presented a mean AFIV of 398.94 ± 0.71 g (N = 6,685), whereas AB individuals showed slightly lower values (396.94 ± 2.08 g; n = 662). Although the BB genotype was rare (n = 14), these animals exhibited a higher mean AFIV (419.98 ± 15.2 g), suggesting a potential additive or genotype-specific effect.

For AOTD in Landrace pigs, the SNP near *NKX2-6* gene (SNP position: 32220) demonstrated a gradual decrease in average occupation time across genotypes, with AA animals showing higher values (135.95 ± 2.29 s; N = 375) compared with AB (133.72 ± 0.88 s; N = 2,440) and BB individuals (132.15 ± 0.65 s; N = 4,546), indicating a consistent genotype-dependent trend. Similarly, for AOTD in Yorkshire pigs, the SNP located near *CPAMD8* gene (SNP position: 6648) revealed modest phenotypic differences among genotypes. Mean AOTD values were 135.89 ± 1.24 s for AA (N = 1,954), 133.63 ± 0.96 s for AB (N = 3,325), and 134.41 ± 0.70 s for BB animals (N = 6,260), suggesting small but detectable genotype-associated variation. These genotype–phenotype visualizations provide an intuitive representation of allelic effects supporting the GWAS associations, although effect sizes remain moderate, consistent with the polygenic architecture expected for feeding behavior traits.

### 3.5 Overlap with known QTL

The overlap between significant SNPs identified in this study and previously reported QTLs was evaluated to provide biological context for the findings of the genome-wide association study. A QTL overlap analysis was performed using the GALLO package (Fonseca et al., 2020), considering a genomic window of ±500 kilobases around each significant SNP, and querying the Pig QTL database (Hu et al., 2022). Genes located within QTL regions containing significant SNPs were retained for downstream functional analyses. In the context of Landrace pigs, overlapping QTLs have been linked to production and carcass-related traits. These traits include, but are not limited to, intramuscular fat percentage, litter weight (number of piglets born alive), loin muscle area, backfat thickness, body weight, abdominal circumference, meat color, and average daily gain (see Supplementary Table S5 for further details). These results suggest that genomic regions influencing feeding behavior may also contribute to growth and body composition traits.

In Yorkshire pigs, SNPs associated with AOTD exhibited an overlap with QTLs linked to loin thickness, Longissimus muscle pH, meat color, cholesterol level, average daily gain, total number of piglets born alive, intramuscular fat percentage, backfat thickness, lean cuts percentage, and C18:0 fatty acid content in Longissimus dorsi (Supplementary Table S5). From these regions, five genes - *KIRREL1, TOX3, TP53BP2, XKR6,* and *CST7* - were selected for downstream functional enrichment analysis using DAVID, as they were located within QTL intervals harboring significant SNPs.

Overall, the QTL overlap analysis indicated that genomic regions associated with feeding behavior traits are enriched for loci previously implicated in economically important production, carcass, and reproductive traits in pigs.

### 3.6 Functional enrichment

**FIGURE 3:**
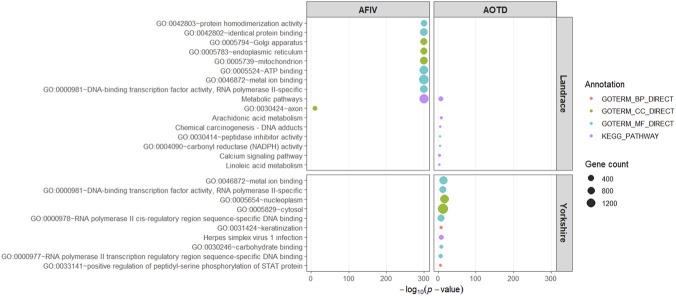
Only significantly enriched GO terms and KEGG pathways obtained from DAVID analysis (Benjamini–Hochberg adjusted p < 0.05) are shown. The top 10 terms per trait and breed are displayed for visualization clarity.

Functional enrichment analysis revealed biological processes and pathways potentially involved in the regulation of feeding behavior traits in pigs. The enriched Gene Ontology (GO) terms and KEGG pathways highlighted molecular functions and metabolic processes associated with energy metabolism, transcriptional regulation, and cellular signaling (Figure 3). For AFIV and AOTD in Landrace pigs, enrichment results indicated significant involvement of metabolic-related pathways, including Metabolic pathways (ssc01100), Arachidonic acid metabolism (ssc00590), and Linoleic acid metabolism (ssc00591). Additionally, GO terms associated with transcription factor activity and peptidase inhibitor activity were significantly overrepresented (Supplementary Tables S6, S7), suggesting regulatory mechanisms influencing nutrient utilization and feeding dynamics.

In Yorkshire pigs, genes associated with AOTD were primarily enriched for cellular component and molecular function categories, including nucleoplasm and cytosol localization, metal ion binding, and DNA-binding transcription factor activity (Figure 3). These findings indicate potential roles of transcriptional regulation and intracellular signaling processes in modulating feeding behavior patterns. Moreover, enrichment of Metabolic pathways (ssc01100) further supports the involvement of energy homeostasis mechanisms, while the Herpes simplex virus 1 infection pathway (ssc05168) likely reflects shared molecular components related to immune signaling and cellular regulatory processes rather than viral response itself. Complete enrichment results are provided in Supplementary Tables S6, S7.

### 3.7 Transcription factors associated with the studied SNPs

**FIGURE 4:**
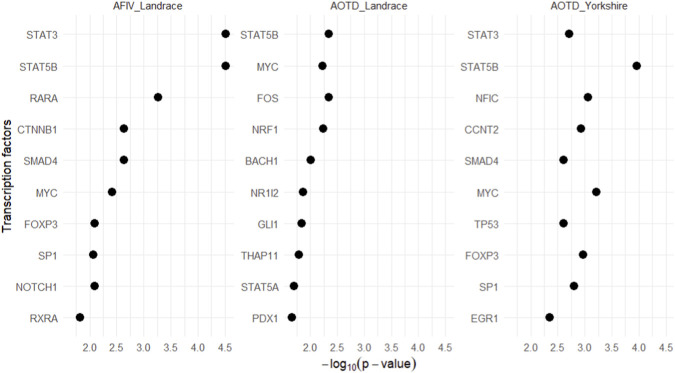
Dot plot showing the top 10 transcription factors associated with the studied SNPs for each feeding behavior trait and breed. Transcription factors are displayed on the y-axis, while the x-axis represents statistical significance expressed as −log10(p-value). Each dot corresponds to one transcription factor.

We employed the Enrichr platform to investigate the regulatory landscape of feeding behavior traits through transcription factor (TF) enrichment analysis based on genes located within GWAS-associated regions. This approach identifies TFs whose target genes are overrepresented in the candidate gene list rather than evaluating whether SNPs are physically located within TF binding sites. The top ten TFs predicted to regulate genes associated with AFIV in Landrace and AOTD in both Landrace and Yorkshire pigs are represented in Figure 4. The identified TFs included STAT3 (Signal Transducer and Activator of Transcription 3), STAT5B (Signal Transducer and Activator of Transcription 5B), RARA (Retinoic Acid Receptor Alpha), CTNNB1 (Catenin Beta 1), SMAD4 (SMAD Family Member 4), MYC (MYC Proto-Oncogene, BHLH Transcription Factor), FOXP3 (Forkhead Box P3), SP1 (Specificity Protein 1), NOTCH1 (Notch Receptor 1), and RXRA (Retinoid X Receptor Alpha). Notably, genes identified in Landrace exhibited the highest enrichment signals for AFIV-associated regions. The complete results are show in Supplementary Tables S8 and Figure 4.

### 3.8 Integrative genomics viewer (IGV) analyses

The IGV analyses were primarily utilized to illustrate the regulatory context of selected key SNPs, interpreting findings based on the functional categories described by Pan et al. (2021). This framework classifies chromosomal regulatory regions according to their epigenetic states and accessibility profiles, employing a combination of histone modification marks, DNA methylation patterns, and chromatin accessibility data (such as ATAC-seq and DNase-seq) to define functional elements like promoters, enhancers, insulators, and repressive regions. By applying this comprehensive annotation framework, we identified significant SNPs located within actively transcribed regions, various classes of active and weak enhancers, and other regulatory elements such as poised promoters. These observations directly support the identification of candidate regulatory variants and their potential roles in gene expression modulation associated with the traits under study.

**FIGURE 5:**
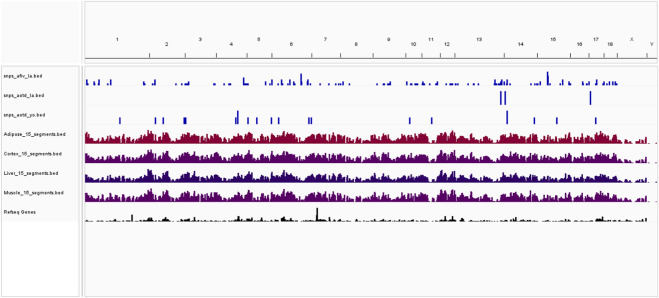
Visualization of statistically significant feeding behavior-associated SNPs (single-nucleotide polymorphisms; blue bars: AFIV in Landrace, AOTD in Landrace, and AOTD in Yorkshire) within tissue-specific chromatin regions. Tissue tracks (adipose, muscle, liver, and cerebral cortex) are color-coded. The files snps_afiv_la.bed, snps_aotd_la.bed, and snps_aotd_yo.bed contain the significant SNPs associated with AFIV in Landrace, AOTD in Landrace, and AOTD in Yorkshire, respectively.

Figure 5 illustrates the overlap between feeding behavior-associated significant SNPs (AFIV in Landrace, AOTD in both Landrace and Yorkshires) and regulatory regions identified by Pan et al. (2021) in adipose, muscle, liver, and cerebral cortex tissues. Significant SNPs are shown as bars along the genome, with chromatin regions color-coded to represent accessibility and epigenetic modification levels. This visualization allows assessment of the distribution of feeding behavior-associated genetic variants within potentially relevant tissue-specific regulatory regions.

Supplementary Figure S3A highlights the SNP rs706356794 (SSC1:7,315,266 Mb), located near *SLC22A2*, as predicted by VEP, a gene potentially influencing AFIV in Landrace pigs. This variant is positioned within a putative regulatory region proximal to *SLC22A2*. Genomic inspection using IGV revealed that rs706356794 overlaps a predicted enhancer region containing transcription factor binding motifs, suggesting a potential regulatory role. The genomic context supports the hypothesis that this SNP may influence transcriptional regulation rather than protein-coding sequence variation. The prioritization of rs706356794 was supported by multiple lines of evidence indicating its potential functional relevance to feeding behavior traits. Notably, the variant is located within annotated regulatory elements, including a poised enhancer in adipose tissue and Polycomb-repressed regions in cortex and muscle, suggesting tissue-specific regulatory effects on nearby genes (Pan et al., 2021; Ngubo et al., 2023). The integration of QTL data, chromatin state maps, and tissue-specific regulatory annotations supports the relevance of rs706356794 as a strong candidate influencing AFIV in Landrace pigs. In summary, rs706356794 was prioritized due to its strategic location in regulatory regions, its proximity to a gene with clear functional relevance to neurotransmitter regulation, and the convergence of evidence from genomic, epigenomic, and functional studies, making it a robust candidate for further functional validation studies.

Chromatin analysis revealed tissue-specific regulatory contexts: in adipose tissue, the before mentioned SNP is near a poised enhancer (E10); in cortex, it is near a repressed Polycomb region (E13); in liver, it is near a medium enhancer with ATAC signal (E7); and in muscle, it is near a weakly repressed Polycomb region (E14). These chromatin state annotations were derived from the ENCODE project, which provides detailed chromatin maps across tissues (ENCODE Project Consortium, 2012). The tissue-specific regulatory contexts observed are consistent with the role of Polycomb repressive complexes in controlling gene expression in a cell- and tissue-specific manner (Ngubo et al., 2023). Together, these findings suggest that rs706356794 may play a regulatory role in AFIV, with effects that vary across tissues.

The significant SNP rs81305085 (SSC14:7,815,388 Mb), located near the *NKX2-6* gene, is situated in a genomic region that may directly influence *NKX2-6* expression, potentially affecting the AOTD trait in Landrace pigs. Chromatin analysis revealed that in adipose and cortex tissues, this SNP is near a bivalent/poised transcription start site (TSS; E12), characterized by both activating and repressive histone modifications (ENCODE Project Consortium, 2012). In liver and muscle, it is located near a repressed Polycomb region (E13), suggesting epigenetic repression by the Polycomb Repressive Complex (PRC) (Ngubo et al., 2023; Supplementary Figure S3B). These tissue-specific chromatin states indicate that rs81305085 may regulate *NKX2-6* expression in a context-dependent manner.

Another highlighted SNP was rs329447474 (SSC2:60,725,486 Mb), located near the *CPAMD8* gene. This SNP, associated with AOTD in Yorkshire pigs, is situated in a genomic region that may directly influence *CPAMD8* expression. Chromatin state analysis revealed that in adipose, cortex, and liver tissues, rs329447474 is near an ATAC island (E11), a continuous region of open chromatin identified by ATAC-seq (Buenrostro et al., 2013; ENCODE Project Consortium, 2012). In muscle, it is located near a weakly repressed Polycomb region (E14), indicating potential epigenetic repression by the Polycomb Repressive Complex (PRC) (Ngubo et al., 2023). The proximity of this SNP to different chromatin states across tissues, together with its association with AFIV in Landrace pigs, suggests a complex, tissue-specific regulatory role (Supplementary Figure S3C).

## 4 Discussion

This study provides three main findings regarding the genetic architecture of feeding behavior traits in pigs. First, GWAS results indicated that a substantial proportion of associated variants are located in regulatory genomic regions, suggesting that transcriptional regulation plays a central role in controlling feeding behavior traits. Second, integrative functional analyses consistently prioritized candidate genes located near key SNPs, particularly *SLC22A2, NKX2-6*, and *CPAMD8*, which showed biologically plausible associations supported by genotype-phenotype comparisons. Third, enrichment and QTL overlap analyses revealed that feeding behavior traits are influenced by complex biological networks involving metabolic pathways, transcriptional regulation, and cellular signaling processes. Together, these findings highlight the polygenic and regulatory nature of feeding behavior traits and provide candidate genomic regions for future functional validation and genomic selection strategies.

The identification of such genomic regions further emphasizes the critical value of assessing feeding behavior traits, which provide insights beyond total feed intake by capturing individual variability in feeding patterns that influence feed efficiency and growth. Traits such as feeding rate, visit frequency, and feeding duration are genetically associated with efficiency indicators, supporting their use as complementary phenotypes in breeding programs aimed at improving productivity and animal welfare (Ding et al., 2018; Santiago et al., 2021; Poullet et al., 2022). The use of automated feeding systems enables large-scale and continuous phenotyping, facilitating the identification of behavioral indicators linked to efficiency and health status (Carcò et al., 2018; Ramonet and Bertin, 2018; Fornós et al., 2022).

The functional annotation of associated variants suggests that feeding behavior traits are influenced by a complex genetic architecture involving both coding and regulatory mechanisms. Although a small proportion of missense variants, the predominance of intronic and regulatory variants highlights the importance of gene expression modulation, post-transcriptional regulation, and long-range regulatory interactions in shaping these traits (Gong et al., 2021). The detection of variants located in untranslated and splicing-related regions further supports the role of RNA processing and microRNA-mediated regulation in feeding behavior, consistent with previous findings in pigs (Mármol-Sánchez et al., 2022).

In line with these insights into regulatory mechanisms, the present study successfully identified variants located in genomic regions of interest that are associated with feeding behavior in pigs. These analyses highlighted the presence of variants near genes such as *TP53BP2* (rs691778017, SSC 10:19824197-19824197) in AOTD in Landrace, *ACSS1* (rs80811001, SSC17:30803219-30803219) in AFIV in Landrace, and *KCNQ4* (rs81258652, SSC6:170366022-170366022) in AOTD in Yorkshire. Furthermore, the functional implications of the identified genes suggest potential mechanisms underlying the observed associations with feeding behavior. For example, the *TP53BP2*, is located near key genes involved in the PI3K-AKT-mTOR signaling pathway, and has been identified as a candidate gene affecting intramuscular fat content in Iberian pigs (Palma-Granados et al., 2023). This suggests that *TP53BP2* may influence energy balance and nutrient utilization through its potential interaction with this critical metabolic pathway. Similarly, *ACSS1*, associated with the metabolism of pyruvate, glyoxylate, and dicarboxylate, has been linked to muscle growth in Tibetan pigs (Nie et al., 2023), further highlighting the importance of metabolic processes in influencing key traits like feed efficiency and growth, which are intrinsically related to feeding behaviour. Finally, the *KCNQ4* has been primarily associated with sensory and neural functions (Kharkovets et al., 2006; Heidenreich et al., 2012). Alterations in this gene may influence feeding behavior–related traits such as AOTD and AFIV through effects on sensory processing, neural regulation, or balance, potentially affecting feeding patterns, digestion onset timing, and variability in feed intake.

These findings, derived from comprehensive variant annotation, consistently revealed that feeding behavior traits are shaped by a complex interplay of genetic influences. Our results not only corroborate the influence of variants in coding regions, such as the missense variants identified, but also emphasize the impact of regulatory variants on gene modulation (Inamo et al., 2024). The identification of SNPs with moderate effects on essential amino acids, as well as variants in enhancers and promoters, provides further evidence of the potential of such variants to contribute to the development of breeding strategies based on integrated functional and genomic data (Supplementary Table S3). Enhancers are non-coding regions of the genome that stimulate the expression of target genes transcribed by RNA polymerase II (RNAPII). These elements can function regardless of their orientation, distance, or position relative to the target gene, and may be situated up to a million base pairs away (Panigrahi and O’Malley, 2021).

Our functional results also revealed variants associated with lncRNA, which are non-coding RNA molecules exceeding 200 nucleotides in length. Several long non-coding RNAs (lncRNAs) were identified near significant SNPs and regulatory regions (Statello et al., 2021; Zhao et al., 2024). These lncRNAs may potentially influence the expression of nearby genes. However, their functional role remains hypothetical and requires experimental validation (Statello et al., 2021; Quinn and Chang, 2016). Given that lncRNAs play crucial roles in regulating gene expression and cellular processes, and variants in these molecules may contribute to the regulation of complex traits, offering potential insights for genetic improvement or therapeutic strategies (Ni et al., 2022). In contrast to messenger RNAs (mRNAs), which are translated into proteins, lncRNAs do not undergo this process. Alternatively, they serve as vital regulators of diverse cellular functions and biological processes (Budak et al., 2020).

In beef cattle, lncRNAs play a pivotal role in traits like feed efficiency and growth, highlighting their potential for genetic improvement and more efficient resource utilization in breeding programs (Alexandre et al., 2020). Karimi et al. (2022) conducted a transcriptome analysis of liver tissue in commercial and native chicken breeds to investigate differences in feed efficiency. Through RNA-Seq and bioinformatics approaches, 2,290 lncRNAs were identified, with 53 differentially expressed between breeds. Functional enrichment analysis suggested that these lncRNAs influence feed efficiency by modulating genes related to lipid metabolism, growth, and energy balance. Co-localization of feed efficiency-related QTLs identified potential candidates, providing significant insights into the genetic regulation of feed efficiency in chickens. These findings collectively underscore the importance of detailed functional analyses to elucidate the regulatory mechanisms that underpin traits of interest and facilitate the application of genomic tools in selection programs.

Consistent with our integrative functional analyses, specific candidate genes were prioritized based on their proximity to key SNPs and their biologically plausible associations with feeding behavior. Among these, *SLC22A2, NKX2-6*, and *CPAMD8* emerged as particularly compelling. The *SLC22A2* gene is involved in the transport of organic molecules and metabolites, which can influence neurometabolic signaling. This gene encodes an organic cation transporter involved in the cellular uptake of several neurotransmitters, including dopamine and serotonin, which are known modulators of feeding behavior and reward-related pathways (Roth et al., 2012). *NKX2-6* plays a role in regulating cardiac development and function but also has implications for gene expression in brain tissues. Finally, *CPAMD8* is associated with immune regulation and developmental processes, which may indirectly impact behavior through interactions between the immune system and the nervous system (Kar et al., 2017; Morrison and Thakur, 2021; Bai et al., 2023; Yang et al., 2024).

Then, the overlap with known QTL identified genomic regions associated with traits such as backfat thickness, *longissimus* thoracis muscle area, meat pH and color, daily weight gain, abdominal resistance (umbilical hernia), and residual feed intake for AFIV in the Landrace population. These QTL have also been reported in other studies, including those by Molinero et al. (2024) on pH and meat color and Lan et al. (2023) on body measurements and reproductive traits in Shaziling pigs. Among these variants, we highlight rs80794948 and rs81234709, that have been demonstrated to influence metabolic traits, such as the proportion of omega-6 fatty acids, and structural characteristics, such as intramuscular fat (Supplementary Table S5). Zeng et al. (2024) identified 12 novel genetic loci associated with growth traits in pigs and reported that the SNP WU_10.2_1_31309038, originally mapped to SSC1:31,309,038 in the Sscrofa10.2 assembly, corresponds to SSC1:27,837,950 in the updated Sscrofa11.1 reference genome, was also within the QTL interval associated with loin muscle area, back fat on the last rib, and average daily gain. This underscores the link between our findings for feeding behavior and growth traits. Fu et al. (2020) identified 32 common SNPs significantly associated with four FE–related traits. Their analysis revealed eight shared QTL regions among these traits, including three regions associated with both ADFI and RFI, indicating a strong genetic overlap. These findings support the interconnectedness of feed efficiency traits in Landrace pigs and provide a reference framework for interpreting the SNPs and QTLs identified in the present study.

In Yorkshire pigs, SNPs located on SSC2 associated with AOTD were particularly relevant. These genetic markers may help optimize feed intake and conversion, potentially improving resource utilization and reducing production costs in breeding programs. Understanding their impact could enhance the development of more efficient and sustainable livestock breeding strategies. These markers can be incorporated into customized SNP panels for genomic selection, enabling the optimization of feeding behavior traits, which may reduce feed costs and enhance the sustainability of pig production.

The functional enrichment analysis of genes harboring our interest genomic regions identified biological pathways and molecular processes associated with the traits of interest (illustrated in Figure 3), and TF analysis (Figure 4) provided further insight into the underlying regulatory mechanisms behind these traits. For instance, the presence of TF such as *STAT3*, *TP53*, and *MYC*, which play central roles in the PI3K-AKT (Liu et al., 2023) and the JAK-STAT (Zhang et al., 2024a) pathways, reinforces the relevance of these processes to behavior and metabolic traits associated with feeding.

The PI3K-AKT signaling is involved in regulating cell growth, energy metabolism, and stress response, and has been shown to interact with key TF like MYC and STAT3. These TF act as central regulators within this pathway, influencing metabolic processes that control feeding behavior, energy expenditure, and feed efficiency. This connection underscores the role of metabolic and signaling networks in modulating feeding behavior traits and highlights their potential impact on improving feed efficiency in livestock breeding (Gil et al., 2026). The identification of EGR1 TF, known for rapid responses to environmental stimuli, suggests a mechanism by which external cues influence feeding behavior. This is supported by the enrichment of fatty acid and lipid metabolism pathways, given EGR1’s role in regulating these pathways and its established link to broiler carcass performance (Ye et al., 2025). These results suggest a connection between metabolic homeostasis, feeding behavior, and transcriptional regulation through shared metabolic pathways.

Further deepening our understanding of transcriptional control over feeding behavior, we found pathways such as amino acid metabolism, regulated by TF like STAT5A*,* are critical in cytokine signaling and impact immunity, disease, and growth. Genetic variants in the STAT3/STAT5A/STAT5B region are associated with inflammatory bowel disease (IBD) (Yang et al., 2024). Also, the identification of BACH1, an important TF for muscle development and adipogenesis (Matsumoto et al., 2016; Zhang et al., 2024b), suggests that a complex interplay where lipid metabolism, immune response, and muscle development collectively influence feeding behavior. Moreover, the presence of TP53, which transactivates the transcription of downstream target genes by binding specifically to their regulatory sequences in response to a variety of cellular stresses, and it is involved in a variety of biological processes such as cell cycle control, apoptosis, aging, differentiation, and DNA repair (Huang et al., 2024), further highlights the intricate connection between cellular repair mechanisms and metabolic adaptation to different dietary demands (Dohi et al., 2008).

Analysis of chromatin regions associated with significant SNPs for feeding behavior traits provided insights into the underlying regulatory mechanisms, revealing distinct epigenetic states and levels of accessibility that suggest specific roles in gene regulation for phenotypic traits such as AFIV and AOTD. The term “chromatin repressed states” refers to genomic regions where the chromatin structure is compacted, limiting access to transcriptional machinery and thereby reducing or silencing gene expression. These states are typically characterized by specific epigenetic marks, such as H3K27me3, which are deposited by Polycomb Repressive Complexes (PRC) and are indicative of transcriptional repression (Ngubo et al., 2023; Pan et al., 2021). In our study, chromatin states were inferred from publicly available epigenomic annotations for pig tissues using the ChromHMM framework, as reported by Pan et al. (2021). The identification of SNPs within or near repressed chromatin regions provides insight into tissue-specific regulatory potential; for example, SNPs located in repressed regions may have limited regulatory impact in that tissue but could be active in others where the chromatin is accessible. Therefore, integrating chromatin state information allows for a more refined interpretation of the functional relevance of SNPs associated with feeding behavior traits in pigs.

The SNP rs706356794, identified in the vicinity of the *SLC22A2* gene, afore mentioned as a putative candidate gene, stands out by exhibiting diverse epigenetic patterns across tissues. In adipose tissue, its proximity to a poised enhancer (E10) suggests a potential regulatory role in gene expression in response to environmental stimuli (Klemm et al., 2019; Wang et al., 2024). Conversely, in the cortex, this SNP is associated with a Polycomb complex-mediated repressor state (E13), suggesting that the *SLC22A2* gene may be functionally silenced in this tissue, though the precise mechanism by which PRCs dampen the expression of active PRC genes remains unclear (Kar et al., 2017). On the other hand, in the liver, its association with a medium enhancer (E7) reflects intermediate accessibility, further suggesting that this SNP modulates gene activity in a tissue-specific manner. These findings highlight the potential influence of epigenetic status on the functionality of SNPs and their effect on the AFIV trait in Landrace pigs. The rs81305085SNP, near *NKX2-6*, also cited as a putative candidate gene, highlights the importance of bivalent/poised TSS regions (E12) in adipose and cortex tissues, where active and repressive histone modifications coexist, allowing for context-dependent gene activation or repression (Yang et al., 2024). In contrast, the association with Polycomb repressor regions (E13) in the liver and muscle suggests epigenetic silencing (Piunti and Shilatifard, 2021). This dynamic regulation of *NKX2-6*, potentially influenced by rs81305085, may contribute to the observed AOTD phenotype in Landrace pigs.

Finally, the SNP rs329447474 (AOTD in Yorkshire), located near the putative candidate gene *CPAMD8*, also showed variable chromatin states. The association with open chromatin regions (E11, “ATAC islands”) in tissues such as the liver and cortex suggests a direct regulatory potential on gene transcription in these tissues. In general, euchromatin, characterized by a more relaxed structure, is associated with more active gene transcription, whereas heterochromatin, which has a more compact structure, contributes to transcriptional repression and genome stability (Morrison and Thakur, 2021). For example, accessible cis-regulatory elements, such as promoters, enhancers, and silencers, located in regions of open chromatin, allow interaction with TF to promote activation or repression of target genes, and this epigenetic control of gene expression plays a crucial role in the regulation of many biological processes, such as development, and different physiological activities in various tissues (Bai et al., 2023). In muscle, however, the SNP was associated with a “weakly repressed Polycomb” state (E14), suggesting that the gene may be less active or functionally silenced in this tissue. In this context, PRCs act as major histone modifiers and play an essential role in maintaining the pluripotent state of embryonic stem cells by repressing key developmental regulators (Boyer et al., 2006). There are two major types of PRCs: PRC1, which is responsible for the monoubiquitination of histone 2A at lysine 119 (H2Aub1) via the ubiquitin ligase RING1A/B; and PRC2, which is responsible for the dimethylation and trimethylation of H3K27 (H3K27me2/3) via the histone methyltransferase (HMT) EZH1/2 (Kar et al., 2017). These findings suggest that they may play a role in shaping the organism’s phenotype.

The distinct chromatin patterns observed underscore the critical need for integrating both genomic and epigenomic data to unravel the regulatory mechanisms underlying complex traits, such as feeding behavior. The analyses of chromatin categories revealed that the significant SNPs identified are not only associated with feeding behavior traits but also exhibit tissue-specific roles, reflecting distinct regulatory states. Togheter, our findings provide a robust foundation for future research focused on tissue-specific gene regulation and the refinement of genomic selection strategies (Liu et al., 2020).

The challenges of this study include the complexity of feeding behaviors, such as AFIV and AOTD, which are influenced by multiple genetic, epigenetic, and environmental factors. Accurate phenotypic data collection is challenging, as individual variability in pigs' feeding behavior requires continuous and rigorous monitoring. Additionally, interpreting data from GWAS can be complex, especially in distinguishing true biological signals from statistical noise (Correia et al., 2014). Understanding the epigenetic regulatory mechanisms is also difficult, as the interaction between regulatory regions, such as promoters and enhancers, and genes needs to be investigated in detail.

For future studies, it would be important to refine phenotypic data collection methods, replicate the results in independent pig populations, and perform functional validation of candidate genes, such as *SLC22A2*, *CPAMD8*, and *NKX2-6*, through silencing or knockout experiments. Epigenomic profiling should also be further explored, using technologies like ChIP-seq to identify epigenetic modifications in relevant tissues. Furthermore, integrating environmental factors into the analyses and improving computational tools for integrating multi-omics data could provide new insights into the regulation of feeding behavior. Longitudinal studies to track changes over time, as well as expanding the research to other species, could offer a broader understanding and practical applications in improving pig production and animal welfare.

Although GWAS provides a powerful framework for identifying genomic regions associated with complex traits, detected associations may include false positives due to population structure, linkage disequilibrium, and sampling effects. Therefore, validation of the identified loci in independent populations or through functional approaches is an important step to confirm biological relevance (Visscher et al., 2017).

In conclusion, our results revealed genes and QTLs associated with the feeding behaviour studied traits, including transcription factors such as STAT3, STAT5A, RARA, and MYC, which are known to bind promoters and enhancers of actively expressed genes. In parallel, candidate genes like *SLC22A2*, *CPAMD8*, and *NKX2-6* were also identified, offering valuable insights into the epigenomic regulatory regions located within or near the associated QTLs. Together, these findings enhance our understanding of the regulatory mechanisms that influence AFIV and AOTD traits in pigs.

## Data Availability

The datasets used for this study are not publicly available because they belong to a swine breeding company and the data is commercially sensitive. Requests to use the data for research purposes should be directed to AcuFast Swin (https://www.acufastswine.com/en), or to Aline Silva Mello Cesar, alinecesar@usp.br and Andre C. Araujo, andre.araujo@acufastswine.com.
