# Supplementary material for: Unraveling quantitative trait loci (QTL) overlapping epigenomic regulatory regions associated with feeding behavior in pigs
Source: Front Genet. 2026 Apr 10;17:1779847. doi: 10.3389/fgene.2026.1779847 (PMC13105475; doi:10.3389/fgene.2026.1779847)
Supplement: Supplementary file 2 [file DataSheet1.docx]

***Supplementary Material***

# Supplementary Data

# Supplementary Figures and Tables

## Supplementary Figures


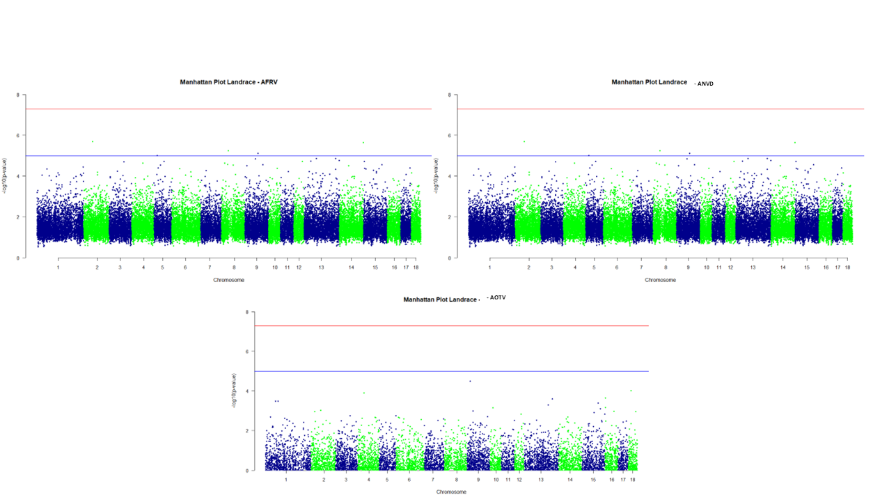


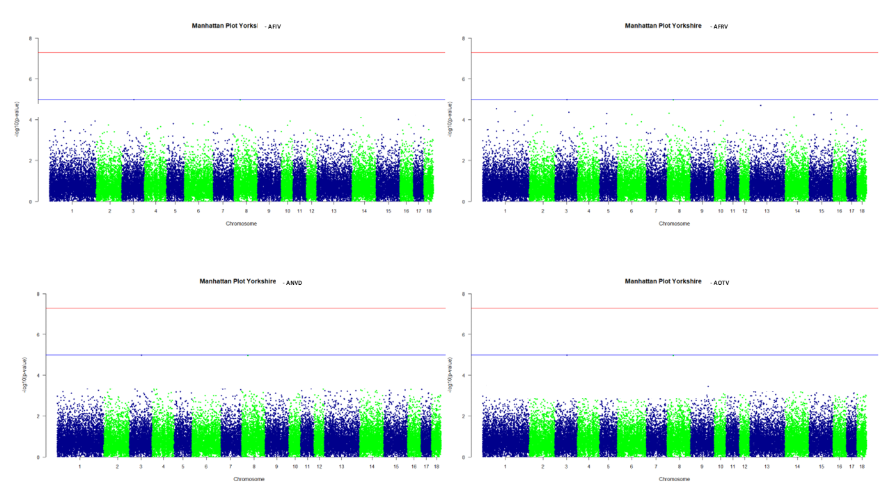


**Supplementary Figure 1.** Manhattan plots of genome-wide association studies for AFRV, ANVD, and AOTV in Landrace pigs, with a Bonferroni-corrected −log10(p-value) significance threshold of 5.91, and Manhattan plots of genome-wide association studies for AFIV, AFRV, ANVD, and AOTV in Yorkshire pigs, with a Bonferroni-corrected −log10(p-value) significance threshold of 5.95.


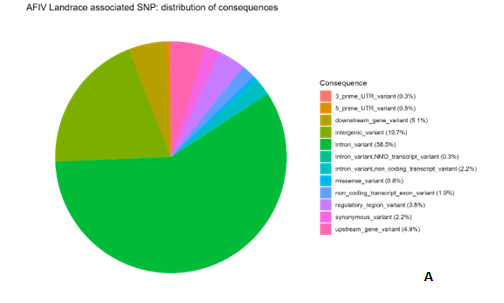


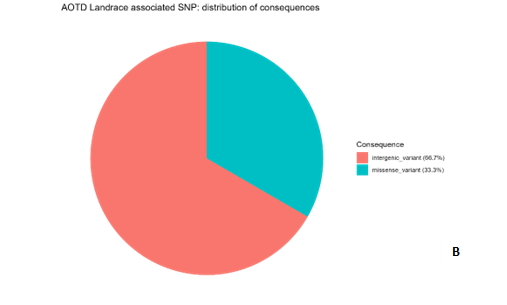


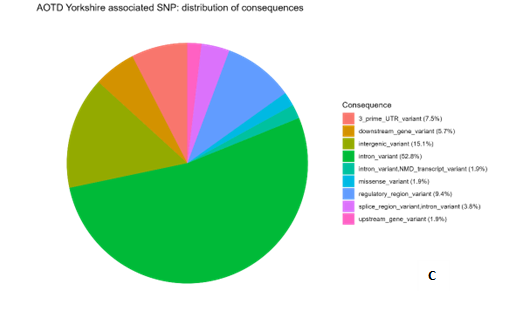


**Supplementary Figure 2.** Distribution of Variant Effect Predictor (VEP) annotated consequences for significant SNPs associated with AFIV_LA (A), AOTD_LA (B), and AOTD_YO (C) traits.


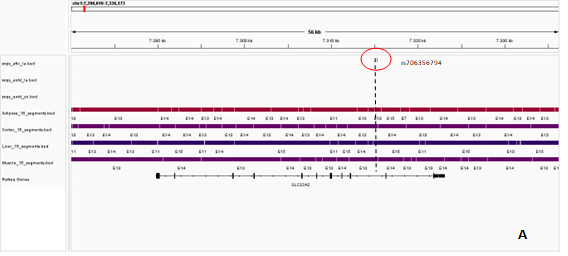


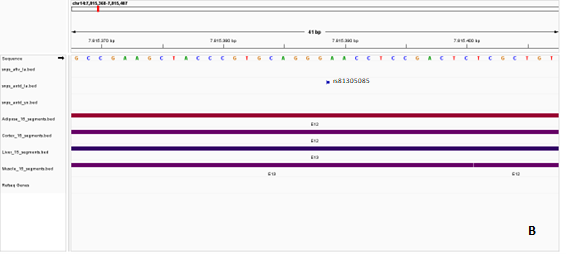


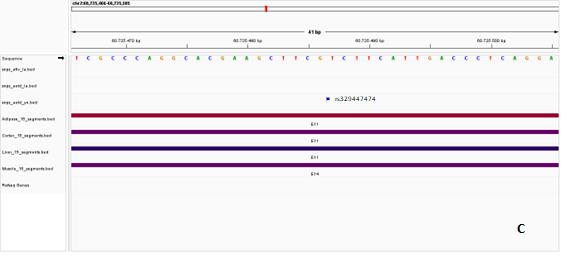


**Supplementary Figure 3.** IGV screenshots showing genomic regions containing statistically significant single-nucleotide polymorphisms (SNPs) identified based on genome-wide association studies (GWAS) for feeding behavior traits AFIV_LA (A), AOTD_LA (B), and AOTD_YO (C), respectively. These SNPs were visualized in conjunction with regulatory data (e.g., ATAC-seq peaks and histone modifications) from adipose, cortex, liver, and muscle tissues based on the dataset published by Pan et al. (2021).

**2.2 Supplementary Table**

**Supplementary Table S1.** Summary of significantly associated SNPs, including chromosomal location, SNP count, and p-values for AFIV, AOTD Landrace, and AOTD Yorkshire.

**Supplementary Table S2.** Summarizes the top 1% of windows, explaining the highest proportion of variance for feeding behavior traits in the Yorkshire and Landrace breeds.

**Supplementary Table S3.** Functional annotation of selected SNPs based on genomic position and consequence for AFIV, AOTD Landrace, and AOTD Yorkshire.

**Supplementary Table S4.** Genomic annotation of selected SNPs with predicted functional consequences for AFIV and AOTD Landrace breed and AOTD Yorkshire breed.

**Supplementary Table S5**. QTLs overlapping or near significant SNPs, including trait category, genomic coordinates, and reference sources from the Animal QTLdb for AFIV and AOTD Landrace breed and AOTD Yorkshire

**Supplementary Table S6.** Functional enrichment analysis of candidate genes based on GO and KEGG pathway annotations for AFIV and AOTD Landrace breed and AOTD Yorkshire breed.

**Supplementary Table S7**. TOP 10 Functional enrichment analysis of candidate genes based on GO and KEGG pathway annotations for AFIV and AOTD Landrace breed and AOTD Yorkshire breed.

**Supplementary Table S8.** Enrichment analysis of transcription factors associated with candidate genes for AFIV and AOTD in the Landrace breed and AOTD in the Yorkshire breed.
